# Predicting Quality of Life Changes in Hemodialysis Patients Using Machine Learning: Generation of an Early Warning System

**DOI:** 10.7759/cureus.1713

**Published:** 2017-09-25

**Authors:** Shoab Saadat, Ayesha Aziz, Hira Ahmad, Hira Imtiaz, Zara S Sohail, Alvina Kazmi, Sanaa Aslam, Naveen Naqvi, Sidra Saadat

**Affiliations:** 1 Department of Nephrology, Shifa International Hospital, Islamabad, Pakistan; 2 Medicine, Aga Khan University Hospital, Karachi, Pakistan; 3 Medicine, Shifa International Hospital, Islamabad, Pakistan; 4 Medicine, Shifa College of Medicine, Islamabad, Pakistan; 5 Medicine, Amna Inyat Medical College, Lahore, Pakistan; 6 Medicine, Rawalpindi Medical College, Rawalpindi, Pakistan

**Keywords:** machine learning, classification tree, naïve bayes, hemodialysis, prediction, quality of life

## Abstract

Objective

To predict changes in the quality of life scores of hemodialysis patients for the coming month and the development of an early warning system using machine learning

Methods

It was a prospective cohort study (one-month duration) at the dialysis center of a tertiary care hospital in Pakistan. The study started on 1^st^ October 2016. About 78 patients have been enrolled till now. Bachelor of Medicine and Bachelor of Surgery (MBBS) qualified doctors administered a proforma with demographics and the validated Urdu version of World Health Organization Quality Of Life-BREF (WHOQOL-BREF). It was to be repeated after one month to the same patient by the same investigator. Simple statistics were computed using SPSS version 24 (IBM Corp., Armonk, NY) while machine learning was performed using R (version 3.0) and Orange (version 3.1).

Results

Using machine learning algorithms, two models (classification tree and Naïve Bayes) were generated to predict an increase or decrease of 5% in a patient’s WHOQOL-BREF score over one month. The classification tree was selected as the most accurate model with an area under curve (AUC) of 83.3% (accuracy: 81.9%) for the prediction of 5% increase in QOL and an AUC of 76.2% (accuracy: 81.8%) for the prediction of 5% decrease in QOL over the coming month. The factors associated with an increase of QOL by 5% or more over the next month included younger age (<19 years) and higher iron sucrose doses (>278mg/month). Drops in psychological, physical, and social domain scores lead to a decrease of 5% or more in QOL scores over the following month.

Conclusion

An early warning system, dialysis data interpretation for algorithmic-prediction on quality of life (DIAL) was built for the early detection of deteriorating QOL scores in the hemodialysis population using machine learning algorithms. The model pointed out that working on psychological and environmental domains, in particular, may prevent the drop in QOL scores from occurring. DIAL, if implemented on a larger scale, is expected to help patients in terms of ensuring a better QOL and in reducing the financial burden in the long term.

## Introduction

Dialysis patients usually have a long commitment to a certain lifestyle. This, in turn, has a significant impact on their quality of life (QOL), irrespective of the modality used [1]. Several factors, such as environmental, social, psychological, financial, and physical, play an important role in determining the QOL that an individual enjoys [1-3]. Several studies have been carried out worldwide with the purpose of identifying the most significant correlates with a better QOL [4-5]. Since there has been no study specifically aimed at the most important predictors of QOL in order of their strength of association using modern machine learning techniques, the purpose of this study is to produce an early warning system, dialysis data interpretation for algorithmic-prediction on quality of life (DIAL), using machine learning to predict a change in QOL in a hemodialysis patient over the coming month. This will be helpful in directing resources toward the high-risk population group.

## Materials and methods

This was a prospective cohort study (of six months’ duration) at the hemodialysis unit of a tertiary care center in Pakistan. It included all the consenting patients who are more than 15 years of age, diagnosed with end-stage renal disease (ESRD) for more than a year, have been on a certain hemodialysis regimen (twice or thrice weekly) for at least three months, and don’t have any disability in communication. All those who did not fulfill the inclusion criteria, patients with a known psychological disorder, patients admitted to critical care units, and patients who had recently (within the last three months) switched from one hemodialysis regimen to the other were excluded from the study. Patients were included by non-probability convenience sampling. Permission for commencement was taken from the local ethics committee. The study started on 1^st^ October 2016.

A total of 78 patients were enrolled. An MBBS qualified doctor administered a proforma with demographic questions and the validated Urdu version of World Health Organization Quality of Life-BREF (WHOQOL-BREF) by Khan MN et al. [6]. WHOQOL-BREF Urdu has already been validated for the hemodialysis population in Pakistan; thus, it was a fitting choice for QOL assessment. WHOQOL-BREF Urdu has 26 questions. Question one asks about an individuals’ overall perception of QOL and question two is about the overall perception of health. The remaining questions pertain to four major domains of life, i.e., physical health, psychological health, social relationships, and the environment. All domains have different raw score ranges; for uniformity, all raw scores were transformed to the 4–20 range according to WHO guidelines. Higher scores show a better QOL. Scores from all the four domains were later combined into one final QOL score. The questionnaire was administered at the start of the study on day zero, then repeated after one month to the same patient by the same investigator. The outcome variable was the amount of change in the total QOL score (delta QOL) over the coming month. The predictor variables were age, gender, income per month, iron sucrose dose per month, and total QOL score at the beginning of the study. Other variables as predictors included changes over the coming month for individual domain scores, hemoglobin, and serum albumin. A first interim analysis was performed on 15^th^ January 2017. Based on the results obtained from the first interim analysis, the foundations of an early warning system, dialysis data interpretation for algorithmic-prediction of quality of life (DIAL), were also laid. DIAL’s sole purpose is to make automated monthly data collection of QOL scores and other predictor variables. DIAL is currently in the implementation phase and its impact on the improvement of the clinical and financial aspects of QOL in dialysis patients will be assessed at a later date after the data is collected. Descriptive statistics in the current study were done using SPSS version 24 (IBM Corp., Armonk, NY). Mean and standard deviations were used to describe continuous variables like age and QOL scores, while percentages and frequencies were used to describe categorical variables. Machine learning was performed using R (version 3.0) and Orange (version 3.1) [7].

## Results

A total of 78 patients were included in the interim analysis. The mean age in years was 51.00 (SD=20). Males comprised 53.8% (42/78) of the total population. The mean duration of hemodialysis was 41.40 months (SD=28.90). The mean albumin levels at the start and end of the one-month period were 3.61 g/dl (SD=0.52) and 3.63 g/dl (SD=0.53), respectively. The means of the total QOL scores at the beginning and end of the one-month study period were 57.6 (SD=10.33) and 59.3 (SD=10.24), respectively, as seen in Table 1.

**Table 1:** Descriptive details of variables included in the analysis QOL: quality of life; DOM: domain

	Overall		Male		Female	
Variables	Mean	SD^1^	Mean	SD	Mean	SD
Age (years)	51.00	20.00	54.00	19.00	47.00	22.00
Duration on hemodialysis (months)	41.40	28.90	47.50	31.30	34.30	24.50
Albumin (g/dl) - start	3.61	0.52	3.66	0.55	3.56	0.48
Albumin (g/dl) - end	3.63	0.53	3.70	0.56	3.56	0.49
Hemoglobin (g/dl) - start	10.42	1.70	10.56	1.84	10.25	1.80
Hemoglobin (g/dl) - end	10.06	1.55	10.07	1.85	10.04	1.13
Change^2^ in DOM1 - Physical	0.40	2.68	0.33	3.08	0.48	2.15
Change^2^ in DOM2 - Psychological	1.01	2.75	1.62	2.91	0.30	2.39
Change^2^ in DOM3 - Social	-0.22	3.51	-0.16	3.89	-0.30	3.08
Change^2^ in DOM4 - Environmental	0.53	2.71	0.68	3.11	0.35	2.19
Change^2^ in QOL	1.71	7.65	2.47	8.59	0.82	6.39
Total QOL score - start	57.61	10.33	58.98	11.06	56.02	9.32
Total QOL score - end	59.32	10.24	61.44	9.05	56.84	11.11
^1^ Standard deviation						
^2^ Change observed over the past month						

A series of student t-tests were carried out to find whether a similar difference exists among genders, as shown in Table 2. It showed there was a significant difference in the mean scores of psychological domains and the overall QOL score among males and females. Males had better relative scores.

**Table 2:** Student T-test on differences in QOL domain scores among males vs females undergoing hemodialysis QOL: quality of life; DOM: domain

Variables	Mean Difference	Sig. (2-tailed)
DOM1 - Physical	-0.9	0.208
DOM2 - Psychological	-1.6	0.044
DOM3 - Social	-1.1	0.085
DOM4 - Environmental	-1.1	0.083
Total QOL Score	-4.6	0.047
Change in QOL Score	-1.60	0.35

A linear regression model was then (p<0.000, r2=0.418) fit. Age, gender, income per month, number of months on hemodialysis, and changes in values for variables like serum albumin, potassium, calcium, phosphate, and hemoglobin were used as predictors for model development. The overall change in total QOL score was selected to be the outcome variable. The model showed monthly income (p<0.000) and serum albumin (p<0.000) to be positively and significantly associated with better QOL, as shown in Table 3.

**Table 3:** Linear regression analysis - coefficients table QOL: quality of life

Change in variable	B^1^	Sig.^2^
Income per family	4.52	<0.000
Albumin	8.14	<0.000
Age	-0.08	0.153
Calcium	-1.55	0.270
Months on HD	-0.04	0.310
Hemoglobin	0.56	0.400
Gender	1.45	0.492
Phosphate	0.31	0.548
Potassium	-0.45	0.682
(Constant)	27.35	0.029
Dependent variable: Positive change in total WHOQOL-BREF score ^1^ Beta coefficient ^2^ Level of significance (P-value)		

**Figure 1:**
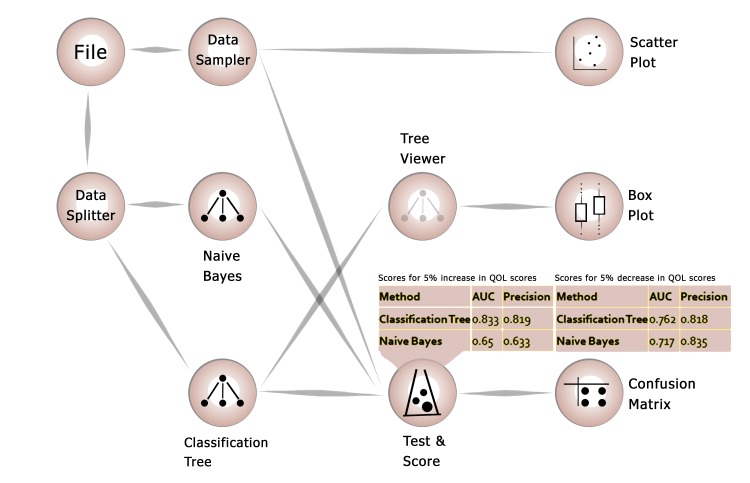
Machine learning algorithm in use with confusion matrices shown for both prediction models (increase or decrease of 5% in QOL score) QOL: quality of life

**Figure 2:**
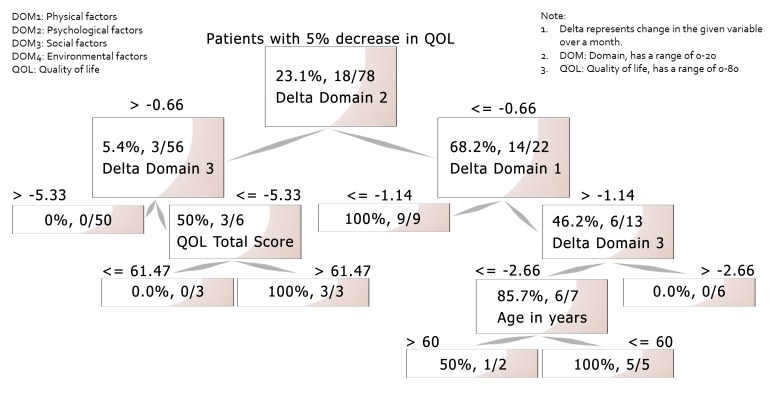
Classification tree: factors associated with decrease in QOL scores by 5% or more QOL: quality of life

Using machine learning algorithms (Figure 1), two models (classification tree and Naïve Bayes) were generated to predict an increase or decrease of 5% in a patient’s WHOQOL-BREF score over one month. The classification tree was selected as the most accurate model with an area under curve (AUC) of 83.3% (accuracy: 81.9%) for the prediction of 5% increase in QOL and an AUC of 76.2% (accuracy: 81.8%) for the prediction of 5% decrease in QOL over the coming month. The factors that were associated with an increase in the QOL score by 5% over the next month were a positive change in domain four (environmental), a total QOL score of <65 at the beginning of the cohort study, age less than 19 years, and iron sucrose doses >278mg/month. The factors associated with a decrease of 5% (Figure 2) in the QOL score over the following month included a decrease in domains two (psychological), one (physical), and three (social), and a greater than 61 total QOL score at the start of the cohort study in order of their importance.

## Discussion

Hemodialysis patients represent a special set of population. After hearing the diagnosis of end-stage renal disease (ESRD), many patients undergo some level of depression [8]. There are physical, social, and psychological impacts on their life, which are reflected in their overall QOL [9-10]. There has always been a need to identify patients at high risk of dropping QOL scores and working on specific domains to aid recovery.

In a tertiary care center of Islamabad, using the WHOQOL-BREF Urdu questionnaire, we collected data regarding the most significant factors that might influence QOL scores in hemodialysis populations. Using modern machine learning methods, we succeeded in building a prediction model that can forecast a change in QOL score in either direction, one month in advance. There have been many studies showing the factors associated with changes in QOL [11]. One of the earlier studies performed in the local population showed that unemployment and psychiatric disease were independently and significantly associated with lower scores of QOL in the dialysis population [12]. To our knowledge, this is the first instance of using modern data analytic techniques to this problem. There is no example of generating an early warning system like DIAL, which may be used as a monthly surveillance system, in the long run, to assess and shortlist patients with the highest risk of having a drop in QOL scores in the coming month. The implementation of such a system is present in other fields [13] and is found to have significant and positive impacts on the financial and clinical aspects of patient management [14].

We also found that domain four (environmental domain) was positively associated with better QOL scores. This is consistent with some earlier studies as well [15]. In an earlier study, age was also found to be significantly associated with good QOL scores [16]. This is also evident in our study. Higher doses of iron sucrose have been given in dialysis patients to replete iron stores [17]. In our study, higher iron replacement doses (>278 mg per month) were found to be associated with better QOL scores. Since the maximum dose given was 800 mg per month intravenously, we could not ascertain whether doses greater than 800 mg are associated with a negative change in QOL scores or not. Other studies have found optimum iron replacement doses in terms of clinical improvement in hemoglobin and, thus, indirectly improving clinical symptoms [18].

Among the patients who suffered a negative change in QOL scores, changes in the psychological, physical, and social domains were the most important contributors. Older patients, but younger than 60 years of age, were more prone to a negative change in QOL scores over the coming month. This may be because these middle-aged patients feel limited and restricted earlier in their life due to dialysis, leading to a greater burden of psychological problems when compared to older (>60) and younger (<30) populations. The relation of increasing age with QOL scores has already been shown in an earlier study [16]. Our study also showed that males had better overall QOL and psychological domain scores when compared to females. The findings were statistically significant but unadjusted for other covariates.

There were also a few limitations in our study. It was an observational study conducted on the local Pakistani population. Also, most of the questions asked regarding QOL were subjective measures of one’s own perception. Since we used the validated questionnaire for our population in their own native language, this factor is expectedly addressed to the maximum possibility. Also, this is an interim report on the ongoing project, which is expected to be completed at the end of 2019. Some of the candidate covariates that are not assessed in the interim analysis but will be used in the final report include serum iron, total iron binding capacity (TIBC), usage of any supplementary medicine/multivitamins, diet regimens, history of receiving any psychological or physical therapies, and so on. Despite the smaller sample size and convenience sampling, the significant values of AUC and a high accuracy suggest a very stable and highly dependable prediction and surveillance system. This leads our team to move on to the publication of the interim results.

Currently, the DIAL screening/surveillance system has been implemented in our institution. We expect to find out if DIAL helps in reducing the long-term financial burden on dialysis patients. The use of machine learning techniques in the health sector will help doctors make smart decisions. This is expected to help doctors in the efficient management of their patients with more confidence.

## Conclusions

In this study, we built an early warning system, referred to as DIAL, for the early detection of a deteriorating QOL score in the hemodialysis population using machine learning algorithms. This model was able to identify a subset of the hemodialysis population at the highest risk of this deterioration with an AUC of 83.3%. The model also suggested working on the psychological and environmental domains, in particular, to prevent this drop from occurring. DIAL, if implemented on a larger scale, is expected to help patients in terms of ensuring a better QOL and a reduction in the financial burden in the long term.
